# Satisfaction of Tuberculosis Patients with Directly Observed Treatment Strategy under Pakistan Health Care Policy: A Mixed-Method Study

**DOI:** 10.3390/healthcare10122529

**Published:** 2022-12-14

**Authors:** Chitralada Chaiya, Sanaullah Panezai, Shahab E. Saqib, Muhammad Ashraf

**Affiliations:** 1 College of Politics and Governance, Mahasarakham University, Maha Sarakham 44150, Thailand; 2 Department of Geography and Regional Planning, University of Balochistan, Quetta 87300, Pakistan; 3 Directorate of Commerce Education and Management Sciences, Higher Education Department Khyber Pakhtunkhwa, Peshawar 25000, Pakistan; 4 Department of Disaster Management and Development Studies, University of Balochistan, Quetta 87300, Pakistan

**Keywords:** tuberculosis, patient satisfaction, health behavior, services, acceptability of health care, Pakistan

## Abstract

(1) Background: Patients’ satisfaction is based on the perceived health care services of individuals and is influenced by the level of care provided by the health care system. It is often based on the patients’ expectations of care and self-assessment of their experiences. The success of the Directly Observed Treatment Strategy (DOTS) also depends on the quality of health care provided at the Tuberculosis (TB) centers, which can be evaluated by satisfaction levels of the patients. (2) Methods: A tuberculosis facility-based cross-sectional study was carried out in Khyber Pakhtunkhwa province in Pakistan. A mixed-method approach was adopted for data collection. An interviewer-administered questionnaire was used for quantitative data collection from 269 patients who were registered at 11 TB centers and private clinics. Qualitative data were collected through 20 in-depth interviews, 15 key informant interviews, and a focus-group discussion. Binary logistic regression was employed for analysis of the data. (3) Results: More than half of the respondents (63.94%) were satisfied with the DOTS strategy. A high percentage of patients were dissatisfied with the availability of safe water, waiting space for patients, waiting time, privacy, and the processing of appointments. Results from the binary logistic regression showed that gender (AOR = 2.21, CI 1.07–4.58, *p* = 0.033), marital status (AOR = 3.12, CI 1.45–6.73, *p* = 0.004), employment status (AOR = 5.22, CI 2.44–1.21, *p* = 0.000), home ownership (AOR = 3.82, CI 1.94–7.54, *p* = 0.000), literacy (AOR = 2.17, CI 1.11–4.25, *p* = 0.023), households’ main occupation (AOR = 4.42, CI 1.12–17.38, *p* = 0.033), and level of income (AOR = 2.39, CI 1.13–5.04, *p* = 0.023) were the significant factors affecting satisfaction levels of the patients. (4) Conclusion: There are a number of areas that need improvement for successful TB eradication. Significant work is required to improve the quality of TB care in these specific areas from the patients’ perspective. For instance, female health workers’ involvement in the DOTS program can solve the problems of female respondents in rural areas. Improving the infrastructure facilities at the TB centers, allocation of doctors and nurses at the rural health centers would result in positive outcomes of the DOTS in Pakistan as well as in other developing countries.

## 1. Introduction

The global population is 7.7 billion, with one-fourth (1.7 billion) believed to be latently infected with tuberculosis [1,2]. Pakistan ranks fifth among the high burden countries and accounts for about 61% of the TB burden in the East Mediterranean Region [2]. Each year, an estimated 420,000 new cases of TB emerge and half of these are sputum smear positive. Globally, Pakistan is also estimated to have the fourth highest prevalence of multidrug-resistant TB (MDR-TB) [1,2]. The World Health Organization and national governments are consistently trying to tackle and control tuberculosis (TB). Global efforts to control TB were reinvigorated in 1991, when the World Health Assembly (WHA) through a resolution recognized TB as a major global public health problem. Two targets for TB control were established as part of this resolution—detection of 70% of new smear positive cases, and a cure rate of 85% of such cases by the year 2000. In 1994, the internationally recommended control strategy, later named Directly Observed Treatment Short Course (DOTS) program was launched [3]. In addition, the WHO set Millennium Development Goals (MDGs) to control TB, with a target of 70% case detection and 85% cure rate in the DOTS program by the year 2015 [4]. Its key components include the following: government commitment; case detection by predominantly passive case finding; standardized short-course chemotherapy for, at least, all confirmed sputum smear-positive cases; providing proper case management conditions; a system for regular drug supply; and a monitoring system for program supervision and evaluation. The DOTS framework has subsequently been expanded, further clarified, and implemented in 182 countries [5]. DOTS implementation has helped countries improve National TB Control Programs (NTPs) and has made major progress in TB control. By the year 2004, more than 20 million patients had been treated in DOTS programs worldwide, and more than 16 million of them were cured. The new target set in Sustainable Development Goals (SDGs) is a 90% reduction in TB deaths by the year 2030, and 95% by 2050 [2]. In addition to this, the Stop TB partnership aimed to reduce the global incidence of the illness to less than one case per million people by 2050 [6]. To achieve this target, between 2015 and 2050, the incidence rate must decrease by an average of 20% annually [7].

Pakistan’s National Tuberculosis Program estimates the default rate for new cases to be less than 4%, but recent studies have found up to 16% [8,9]. Whereas the most recent study by Hameed, et al. [10] reported that in Pakistan, the default rate is 27%, which is very high. The default is influenced by factors that vary by geographic locations, such as economics and health beliefs; therefore, successful interventions require an awareness in local contexts [11,12]. Moreover, due to an inefficient health system and other socioeconomic and cultural reasons, the total patient delays in Sub-Saharan Africa were over ten weeks [13], in Asia around seven weeks [14], in Latin America around six weeks [15], and about 14 weeks in Pakistan [14,16]. Within these challenges, to achieve the target set for 2050, the NTP Pakistan has started a public private partnership (PPP), and involved female health workers in the TB program. According to the WHO report, 5% more people were reported to have TB in health centers in Pakistan [17]. This study further states that institutional support is very important for TB patients. The availability of doctors, nurses, midwives, other practitioners, and experts is highly needed [18,19]. The literature has conclusively shown that delay in seeking health care among TB patients is due to the unavailability of health services. For example, Hino, et al. [20] and Paz and Sá [21] reported that the late diagnosis of TB, as a result of people’s delay in seeking health services at the onset of initial signs and symptoms of the disease, linked with the low availability of these services in comparison to the demands of the people, has contributed to increasing their level of debilitation. Therefore, it is concluded that viability of health services and decentralization of the health system can mitigate the health system delay and delay in treatment [14,16].

For assessing health services delivery around the globe, researchers are using quantitative, qualitative approaches or mixed-methods. In the last two decades, the mixed-method approach is becoming popular [22]. Mixed-methods research is important in low-and-middle-income countries, where only a single method cannot be sufficient to understand the socio-cultural and economic contexts that are essential to assess health systems’ performance and satisfaction level of the patients [22]. Considering the advantages, this study has adopted the mixed-method research design.

Several studies have been conducted in Pakistan to address several issues regarding tuberculosis [19,23]. Nevertheless, to the best of our knowledge, less is known in Pakistan about the TB patients’ satisfaction on TB services delivery (DOTS program). Therefore, this research addresses this gap by conducting a study in the Khyber Pakhtunkhwa province of Pakistan. The study will also be useful in terms of exposing the hurdles in DOTS successful implementation for policymakers in the developing world. Moreover, the study investigates the important predictors of TB patients’ satisfaction on DOTS facilities, which will help other developing countries improve TB management.

## 2. Conceptual Framework

**Figure 1:**
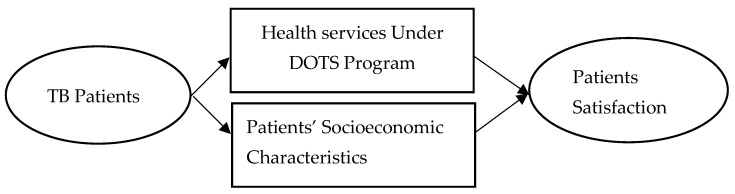
Conceptual framework.

Patients’ satisfaction is based on perceived health care services of individual patients, which is influenced by the level of care provided by the health system [24]. It is often based on patients’ expectations from the health care and self-assessment of their experiences. Patients’ satisfaction plays a major role in utilization of health care services [25], adherence to medication [24], and keeping appointments [24,26]. Patients’ adherence is affected by patients’ satisfaction, which leads to treatment success [27]. Hence, promoting adherence through a patient-centered approach is much more effective than spending resources on tracing defaulters [26]. Other socio-economic factors of the patients are also linked to the satisfaction level. For instance, Onyeonoro, et al. [28] revealed that young patients were comparatively more satisfied than older patients in their study. Likewise, Quintana, et al. [29] and Chimbindi, et al. [30] revealed that marital status affects the patients’ satisfaction level. Onyeonoro, et al. [28] and Mohamed, et al. [24] revealed that higher educational status leads to a higher level of satisfaction, more likely to appreciate information and services provided by DOTS staff, as well as making better informed choices to improve their health. Thus, the success of DOTS depends on the quality of health care provided at the TB centers, which can be evaluated by exploring satisfaction levels of patients [31] as mentioned in Figure 1.

## 3. Materials and Methods

### 3.1. Study Area

**Figure 2:**
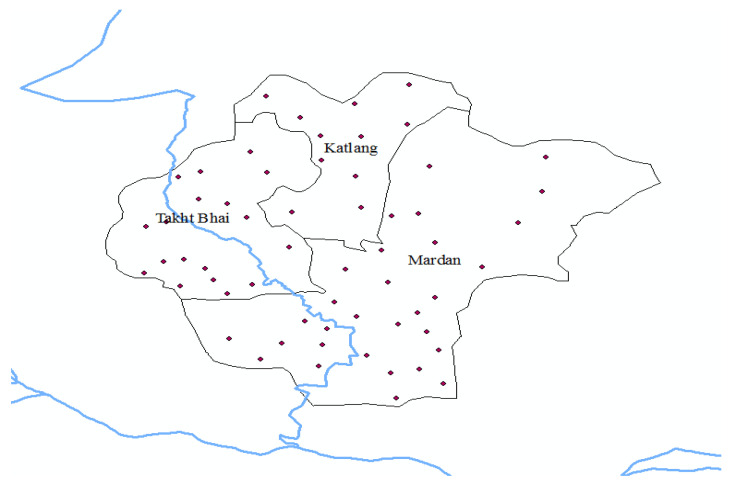
DOTS facilities pre-2012., Dots shows the health centers providing TB care, Blue line shows the river, and black lines are the boundaries of sub districts.

**Figure 3:**
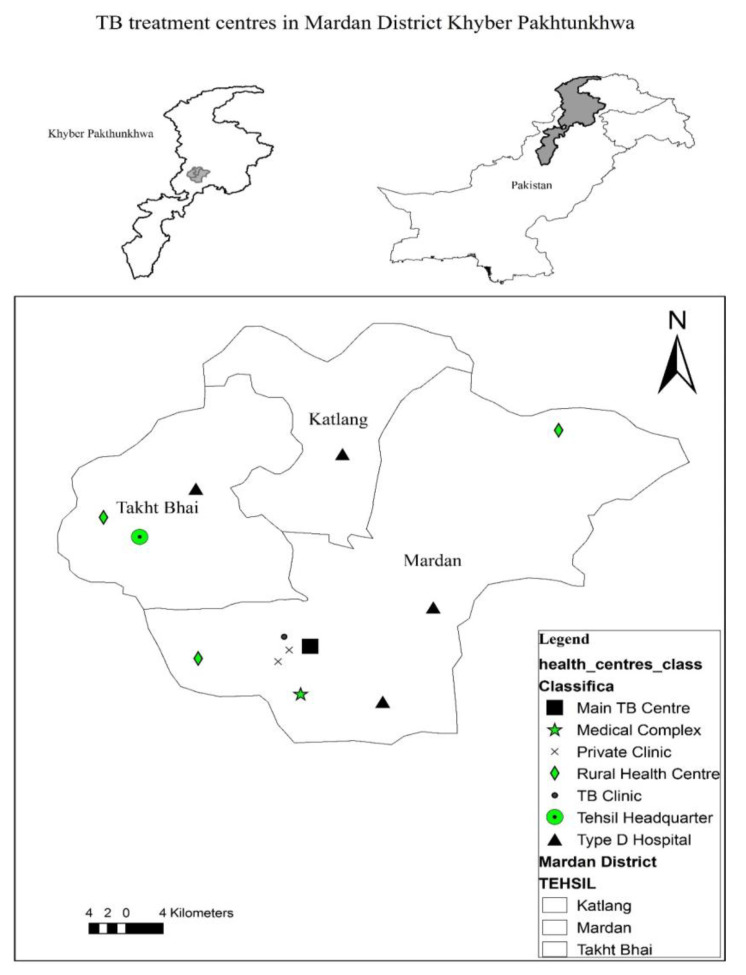
DOTS Facilities Post-2012.

Mardan District is purposely selected as the study area as it is ranked the 2nd largest city of the province. Mardan is situated in the central zone of the province. The district comprises three tehsils: Mardan, Katlang, and Takhtbahi. Before 2012, TB care facilities were provided at main hospitals, Rural Health Centers (RHCs), Category-D hospitals and Basic Health Units (BHUs) in the whole district. The dots in Figure 2 show DOTS facilities scattered around the district. However, after 2012, the DOTS facilities were no longer provided at BHUs. Figure 3 shows the locations of these facilities. The health facilities were centralized as there were many issues related to BHUs. The doctors were not performing duties, and the reporting from these centers was very low [32]. Moreover, the people were still coming to main centers and not utilizing the services in these rural health centers. Therefore, the health centers were more centralized and TB care facilities were confined to few centers.

### 3.2. Study Design

A mixed-method study design is applied in this study. We have interpreted the results of quantitative data analysis with the help of qualitative data to enhance its illustration and clear findings. Further, we adopted the data integration approach in which we integrated qualitative and quantitative data. This study is an explanatory-sequential mixed method in which we have collected data using a questionnaire, supported by key informants interviews (KII), in-depth interviews (IDI), and focus group discussion (FGD). We have not only identified the problems quantitatively, but also from multiple perspectives, developed a complete understanding of the problem, contextualized information, and thus triangulated results [22,33].

### 3.3. Sampling

**Figure 4:**
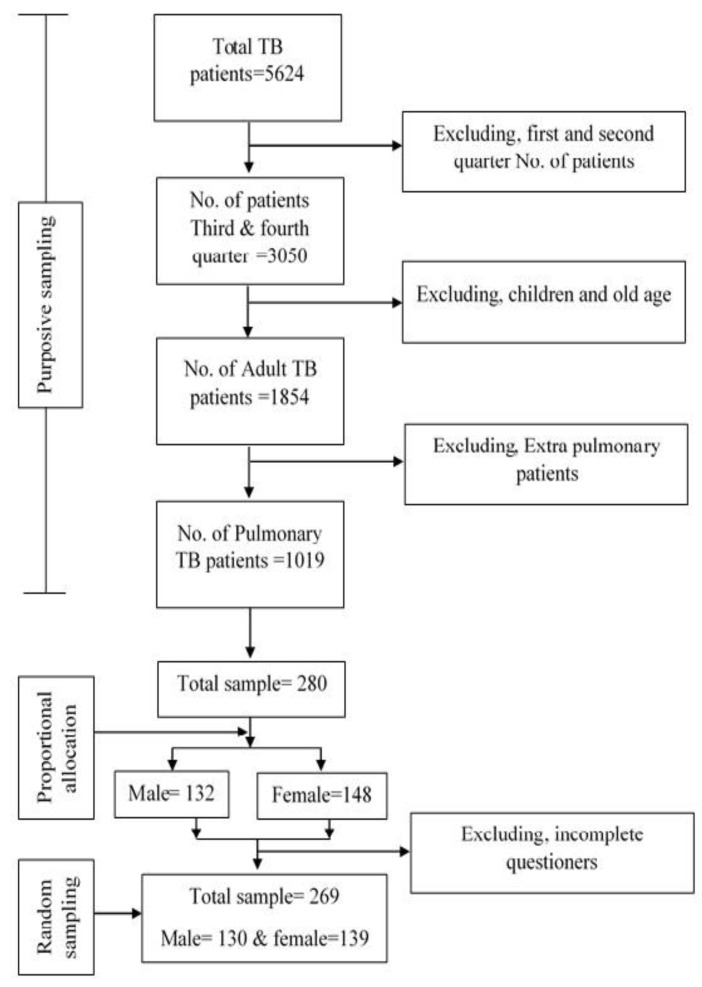
Flow chart of sampling.

Khyber Pakhtunkhwa province was purposively selected among the four provinces due to the high prevalence of TB [34]. Despite the effective Directly Observed Treatment Short Course (DOTS) program in the province, approximately 58,449 new cases of TB were recorded in 2014 [34]. In Khyber Pakhtunkhwa, Mardan District was purposively selected based on its high DOTS population and high TB prevalence. The population is scattered, living in remote areas with a traditional rural lifestyle. The total number of TB patients that were registered at 12 TB centers and private clinics in the district were 5624 in 2016 [35]. Of the patients that were registered in the last two quarters, the third and fourth quarters were taken as the sample population within the ages ≥ 15 years and ≤ 60 years. Excluding children and aged patients, the number of total patients were 3050 in 2016. While in the third and fourth quarters, our sample population was 1854 (Figure 4). Of these patients, we selected the patients that had pulmonary tuberculosis, which amounted to 1019. Of this total population of pulmonary tuberculosis patients, 53% were female and 47% were male patients. The sample size was calculated employing the sampling formula suggested by Naing, et al. [36]:

$$n=\frac{NZ^{2}\times p\times (1-p)}{\left(N-1)d^{2}+Z^{2}\times p\times (1-p\right)}$$

where n = sample size, N = total number TB adult patients (1019), Z = confidence level (at 95 percent level, Z = 1.96), p = estimated population proportion (0.55, this maximizes the sample size suggested by Lwanga and Lemeshow [37]), d = desired error set as 5 percent.

First, the total sample size was allocated to the TB centers by proportional allocation to the number of their patients who were under treatment for the last four weeks. Second, the total sample size was proportionally allocated by gender: 132 male and 148 female patients. The patients with TB who were chosen for follow-up were randomly selected. Among these respondents, 10 females and 6 males did not come during the first two months of data collection. They were interviewed at home in the third month. However, one male and five female respondents were not interviewed due to migration and cultural reasons. At the end of the third month, 131 males and 143 female respondents were interviewed. Five questionnaires were incomplete and had missing values, and could not even be contacted by telephone or mobile phone (4 female and 1 male). Hence, they were dropped from the analysis. Therefore, the total sample was 269, consisting of 130 male and 139 female respondents.

### 3.4. Inclusion Criteria

The respondents in this study were both TB smear-positive and negative under treatment for at least the previous four weeks; and at the time of the interview, had made at least two visits to the TB center. However, we included only those patients who had pulmonary TB.

### 3.5. Exclusion Criteria

We have excluded the extra pulmonary patients from our study population because pulmonary tuberculosis is a highly contagious disease that can spread quickly after infection [38]. The patients below age 15 and above 60 are excluded from our sample population. The children below age 15 and old age above 60 years were excluded due to their inability to evaluate the TB services provided to them. The patients who have completed their treatment were also excluded from the study as it was difficult to trace them.

### 3.6. Data Collection

Data were collected over three months, from 1 November 2016 to 31 January 2017. Five assistants, 3 male and 2 females, who had worked in health surveys with different organizations, were hired to collect data. These assistants were first trained on data collection. However, the principal investigator was present in all stages of data collection. Quantitative data were collected through interviewer-administered questionnaires. The stay-interview procedure was followed from the patients who were under treatment at the centers and came regularly each month to collect medicines and for routine check-up. To minimize the recall bias, we took the following measures: first, we collected data through trained interviewers as discussed, second, our participants were not from very young and very old age, third, we asked questions for the last four weeks, which the patients easily answered. The questionnaire was prepared after an in-depth review of the existing literature [39,40,41,42,43,44], and from experts at the Asian Institute of Technology and TB control program in the district. The questionnaire contained socio-economic and demographic factors of the respondents and 27 items on a 5-point Likert scale for measuring satisfaction level. Of these items, 1 was for the overall satisfaction level on the whole TB treatment course, 11 items were about the structure of the health care system, 11 for the process, and 4 for the outcome of the DOTS program. The questionnaire was initially pre-tested by interviewing 30 respondents. These data were entered in SPSS version-23 (SPSS, Chicago, Illinois, USA) and reliability was tested. The reliability values of overall satisfaction, structure, process, and outcome were 0.957, 0.937, 0.903, and 0.74, respectively.

Qualitative data were collected through 20 in-depth interviews (IDI) conducted with patients and fifteen key informant interviews (KII). For in-depth interviews, with each patient, we spent approximately one hour to 1 and half hours. Written consent was obtained from each participant of the IDI, KII, and FGD. Moreover, data were coded and names of the participants were not shown during data analysis. Key informants comprised of; 1 provincial TB program, 1 District TB Officer (DTO), 1 District Health Officer (DHO), 4 Medical Officers at Rural Health Centers (RHCs), 2 Lab Technician, 2 DOTS facilitators, 1 Store Keeper, 2 Medical Officers from the Basic Health Units (BHUs), and 1 doctor from the Public Private Mix (PPM). These interviews were conducted by the principal investigator. Each interview took 30 to 45 min. Interviews were recorded through audio digital recorder and field notes were taken. Most of the interviews were in the Pushto language. All the recorded data were translated into English with the help of a second author. One focus group discussion (FGD) was conducted in the District TB center, where a majority of the patients were registered. The FGD lasted for 2 h and 30 min. Notes were taken and the discussion was recorded. This group comprised of 7 participants who were provided with pre-prepared questions, such as, what are the strengths and weaknesses of DOTS, what are their experience with the patients, what are the patients’ opinions about the TB control program, how can patients’ satisfaction be improved, and what are the important factors that limit patients’ satisfaction levels.

### 3.7. Validation, Reliability, and Credibility

As discussed above, for instrument validation and reliability, the questionnaire was pre-tested and the Cronbach’s Alpha value was calculated. Moreover, data were first collected by those with experience in health survey data. Second, two-day training was conducted for them, before and after pre-testing the questionnaire. Third, the questionnaires were checked on a daily basis and data collection was strictly supervised by the principal researcher. Fourth, data were collected through different tools. These tools included both quantitative and qualitative: interviewer-administered questionnaire, telephonic interviews, in-depth interviews, key informant interviews, and observations. The results were triangulated and checked. Fifth, respondents were not gender biased and were from diverse backgrounds; moreover, each data collector interviewed one or two respondents at the most, per day, over three months.

### 3.8. Data Analysis

#### 3.8.1. Statistical Tests

We categorized the study variables in different groups. Considering the nature (nominal) of the study variables, Chi-square and Fisher’s exact tests (when a cell has a frequency <5) were used to find the differences between patient groups.

#### 3.8.2. Regression Analysis

For regression analysis, we used binary logistic regression and multi-variate logistic regressions. In this kind of analysis, when the dependent is binary, logistic regression was adopted in several studies. For example, Getahun and Nkosi [40], Portela, et al. [42], and Karim, et al. [45] used the same model for patients’ satisfaction. Therefore, logistic regression is the suitable model and has been used for analysis.

#### 3.8.3. Dependent Variable

The satisfaction level of respondents was measured through a 5-point Likert scale ranging from 1 = very low to 5 = very high. There was a total of 27 items for satisfaction; 1 was for overall satisfaction level, a stand-alone item shown in Appendix A. Taking the mean value as the cut point [40,46], the respondents were divided into two categories: satisfied and dissatisfied. For regression analysis, the dependent variable was binary: 0 = dissatisfied and 1 = satisfied.

#### 3.8.4. Independent Variables

In multivariate logistic regression, we included those variables with a significance level *p* ≤ 0.25 [47]. The unadjusted Odds Ratios (uOR) of 95% CI were calculated through binary logistic regression. For adjusted OR (aOR), we employed a multivariate logistic regression model. Among the socio-economic characteristics, the age was from 15 to 60 years and classified into five classes shown in Table 1. Gender included two categories: female and male. Marital status contained unmarried, married, and deceased wife. Employment status was binary in nature: those who were working were employed, and zero otherwise. Literacy was binary in our case. It included illiterate, those who cannot read or write and literate. In terms of location, we measured whether the patient was from urban or rural areas. Monthly income was measured in national currency (Pakistan Rupees (PKR)). However, we converted it into USD (1 USD= 104.7417 PKR: http://www.sbp.org.pk/ February-2017, accessed on 26 October 2017). In the main household occupation, the servant category is included for those who had formal employment and a fixed monthly salary. In some studies, for example, Zafar [48], it was referred to as white collar jobs.

### 3.9. Operational Definitions of Satisfaction

Patients’ satisfaction means their perceptions, feelings, and emotions on DOTS services delivery. Here, in our case, we measured their perception on Likert-scale (1–5).

## 4. Results

### 4.1. Results of Quantitative Data Analysis

#### 4.1.1. Socio-Economic and Demographic Characteristics of Respondents

**Figure 5:**
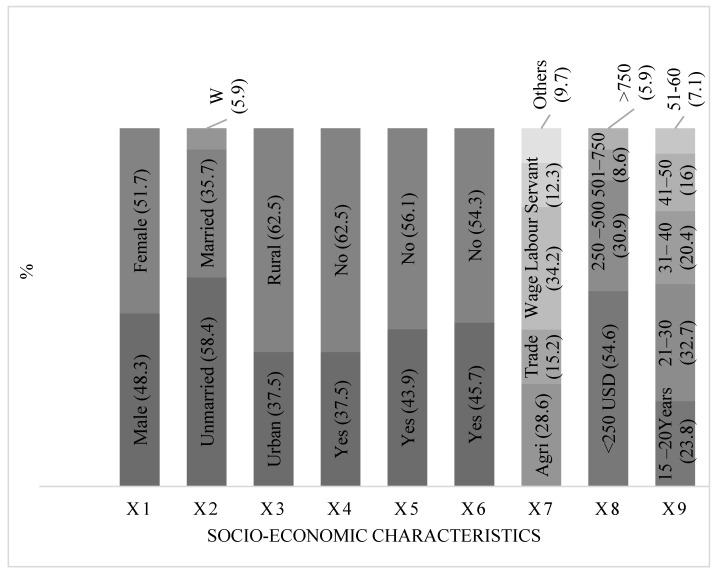
Percentage distribution of respondents. Note: X1 is for Gender, X2 = Marital Status, X3 = Location, X4 = Employment, X5 = House Ownership, X6 = Literacy, X7 = Main Households’ Occupation, X8 = Household Monthly Income, and X9 = Age of Respondents.

Of 269 respondents, there were slightly more females (51.7%) than males (48.3%). Likewise, the higher proportion of TB patients were in rural areas (62.3%); the rest were from urban areas (Figure 1). Of total respondents, more than half (58.4%) were unmarried and young, 15–20 years (23.8%), and 21–30 (32.7%). Among them, 35.7% were married and 15.9% were deceased wife. Employment status included both formal and informal, for which respondents were paid either per month or per day. In this category, 62.5% were unemployed, and similarly, more than half did not own a house. Literate respondents were categorized by those who could read and write; more than half were illiterate. Literacy can be defined as “a person who can read and write a paragraph (3 lines) in their national/regional language with comprehension” (http://www.accu.or.jp/litdbase/policy/pak/, accessed on 24 November 2022). Most of the respondents had agricultural (28.60%) or wage labor (34.20%) as their main household occupation. Likewise, for their level of income, most respondents (54.6%) were from the lower category (<250 USD) of household monthly income (Figure 5).

#### 4.1.2. Satisfaction Level of Respondents

**Figure 6:**
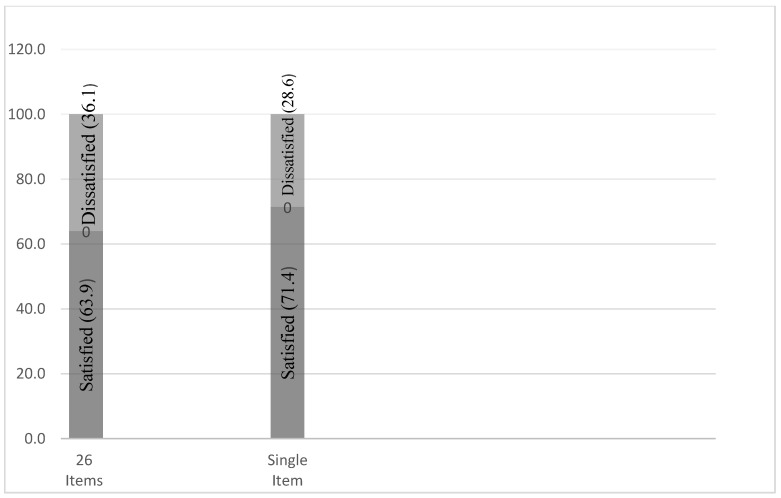
Satisfaction of respondents.

Taking mean value as the cut-point [40,46], the respondents were divided into two categories: satisfied and dissatisfied. According to the stand-alone item, 71.4% of respondents were satisfied with the DOTS program; while from the overall mean of 26 items, the satisfaction was slightly lower (63.9%), as shown in Figure 6.

Respondents’ satisfaction is measured by 26 items for different aspects of the DOTS strategy in the district. Satisfaction values in Table 1 show that overall, the patients’ satisfaction was found to be lower than the neutral value, 3. The combined mean values of 26 items was 2.86, Standard Deviation (SD) = 0.69, and overall Cronbach’s Alpha = 0.943. Likewise, overall satisfaction of the stand-alone item was also lower (Mean = 2.51, SD = 1.11, Cronbach’s Alpha = 0.942) than the moderate value. For satisfaction on structure, the highest satisfaction was 3.58 for two items: access to collect medicine and availability of medicine. The lowest satisfaction was 2.07 for availability of safe drinking water. Regarding DOTS processes, the highest satisfaction was associated with equality for rich and poor (mean = 3.27, SD = 0.06), and skill of health professionals (mean = 2.25, SD = 0.06). However, the lowest satisfaction was for waiting time at the health center, which had a mean = 2.17, SD = 0.07. Reduction in symptoms had the highest mean satisfaction value = 3.55, SD = 0.06 in outcome of the DOTS, and lowest (mean = 2.38, SD = 2.38) for feeling energetic.

**Table 1:** Mean scores and reliability of satisfaction measuring items.

Measuring Items	Very Low n (%)	Low n (%)	Moderate n (%)	High n (%)	Very High n (%)	Mean	SD	Cronbach’s Alpha
Overall Satisfaction 1 Item	53 (19.70)	90 (33.46)	73 (27.14)	40 (14.84)	13 (4.83)	2.51	1.11	0.942
Satisfaction in Structure of DOTS								
Space for Patients	56 (20.82)	108 (40.15)	54 (20.07)	41 (15.24)	10 (3.72)	2.41	0.07	0.940
Availability of Safe Drinking Water	109 (40.52)	75 (27.88)	50 (18.59)	28 (10.41)	7 (2.60)	2.07	0.07	0.943
Availability of Toilets	46 (17.10)	79 (29.37)	71 (26.39)	60 (22.30)	13 (4.83)	2.68	0.07	0.941
Treatment Process	40 (14.87)	90 (33.46)	73 (27.14)	51 (18.96)	15 (5.58)	2.67	0.07	0.940
Access to Medicine	20 (7.43)	22 (8.18)	42 (15.61)	152 (56.51)	33 (12.27)	3.58	0.06	0.940
Privacy at TB Centre	48 (17.84)	108 (40.15)	89 (33.09)	13 (4.83)	11 (4.09)	2.37	0.06	0.941
Laboratory Services	22 (8.18)	20 (7.43)	68 (25.28)	119 (44.24)	40 (14.87)	3.50	0.07	0.941
Availability of Medicines	20 (7.43)	22 (8.18)	42 (15.61)	152 (56.51)	33 (12.27)	3.58	0.06	0.940
Availability of Doctors	34 (12.64)	100 (37.17)	58 (21.56)	66 (24.54)	11 (4.09)	2.70	0.07	0.940
Availability of Equipment	43 (15.99)	102 (37.92)	74 (27.51)	40 (14.87)	10 (3.72)	2.52	0.06	0.942
Availability of Signage/Directions	32 (11.90)	100 (37.17)	70 (26.02)	55 (20.45)	12 (4.46)	2.68	0.06	0.941
DOTS Processes								
Convenience of Service Hours	37 (13.75)	88 (32.71)	57 (21.19)	74 (27.51)	13 (4.83)	2.77	0.07	0.942
Waiting Time	90 (33.46)	93 (34.57)	47 (17.47)	28 (10.41)	11 (4.09)	2.17	0.07	0.941
Appointment Process	30 (11.15)	98 (36.43)	105 (39.03)	27 (10.04)	9 (3.35)	2.58	0.06	0.941
Attitude of Health Personnel	19 (7.06)	65 (24.16)	100 (37.17)	72 (26.77)	13 (4.83)	2.98	0.06	0.941
Care of Health Personnel	21 (7.81)	42 (15.61)	136 (50.56)	55 (20.45)	15 (5.58)	3.00	0.06	0.940
Guidance on Sickness	25 (9.29)	76 (28.25)	63 (23.42)	88 (32.71)	17 (6.32)	2.99	0.07	0.939
Consultation	57 (21.19)	52 (19.33)	69 (25.65)	78 (29.00)	13 (4.83)	2.77	0.07	0.940
Skill of Health Professionals	28 (10.41)	29 (10.78)	72 (26.77)	128 (47.58)	12 (4.46)	3.25	0.06	0.940
Cost of Medicine	30 (11.15)	90 (33.46)	72 (26.77)	56 (20.82)	21 (7.81)	2.81	0.07	0.943
Cost of Transportation	37 (13.75)	82 (30.48)	61 (22.68)	66 (24.54)	23 (8.55)	2.84	0.07	0.942
Equality between Rich and Poor	24 (8.92)	25 (9.29)	93 (34.57)	108 (40.15)	19 (7.06)	3.27	0.06	0.940
Outcome of DOTS								
Symptoms Reduction	18 (6.69)	19 (7.06)	53 (19.70)	155 (57.62)	24 (8.92)	3.55	0.06	0.940
Overall effect of Medicine	26 (9.67)	22 (8.18)	70 (26.02)	130 (48.33)	21 (7.81)	3.36	0.06	0.940
Psychological Effect	44 (16.36)	44 (16.36)	70 (26.02)	95 (35.32)	16 (5.95)	2.98	0.07	0.940
Feeling Energetic	87 (32.34)	62 (23.05)	63 (23.42)	44 (16.36)	13 (4.83)	2.38	0.07	0.941
Total (26 Items)						2.86	0.69	0.943

#### 4.1.3. Comparison of Patients

Respondents were divided into two groups by taking the mean score of satisfaction as a cut-point (Table 2), such as satisfied (n = 172) and dissatisfied (n = 97). Their respective percentages are shown in Figure 2. There was a significant difference between male and female respondent groups in distribution, at a *p*-value = 0.010. The locations, urban and rural, show a significant difference at *p* = 0.01. Males were more satisfied than females with the DOTS strategy. Likewise, marital status, employment, either formal or informal, house ownership, main household occupation, and household level of income were found to be different at *p*-value = 0.000.

#### 4.1.4. Regression Results

The model was a good-fit, as shown by the Hosmer and Lemeshow test values. All variables from the above section were included in the models as their respective *p*-values were ≤0.25. The findings of the model show that males were more satisfied than female respondents with the DOTS strategy (aOR = 2.21, CI = 1.07–4.58, *p* ≤ 0.05). Married patients were 3.126 times more likely to be satisfied, as shown in Table 3. Likewise, satisfaction was higher for employed than unemployed patients (aOR = 5.22, CI = 2.44–1.21, *p* ≤ 0.01). The value aOR 3.82 of the patients with ownership of a house were likely to be more satisfied than patients with no ownership. Literate patients had higher satisfaction than illiterate (aOR = 2.17, CI = 1.11–4.25, *p* ≤ 0.05). From the uOR value, the household’s main occupation as servant was 6.04 times more satisfied than agricultural households, and 4.24 times as shown by aOR. Overall, the patients were from the poor and low-income groups. This is shown in the results that the patients in the income group 250–500 USD were more satisfied than the low-income group, which was <250 USD (uOR = 2.17, CI 1.49–4.93 *p* ≤ 0.01) and (aOR = 2.39, CI 1.13–5.04, *p* ≤ 0.05).

### 4.2. Results of Qualitative Data Analysis

Among participants of the 20 in-depth interviews, 12 (60.0%) were female and eight (40.0%) were males. Ten percent among them were 15–20 years of age, 20.0% from the age group 21–30 years, 30.0% were 31–40 years, 20% were 41–50 years, and 20.0% were 51–60 years. Seventy percent were illiterate, 20.0% had middle level education, and 5.0% had matric and master’s degrees each.

Total key informants (KIs) were fifteen from various sectors working in the DOTS program. The explanation is given in the data collection section. However, the descriptive statistics shows that only one medical officer (6.7%) was female. Among our KIs, 13.3% were in the 21–30 years old age group, 20.0% were 31–40 years, 33.3% were 41–50 years, and 33.3% were 51–60 years. Among them, 6.7% had a higher education, 26.7% had a bachelor, and 66.6% had master’s level education.

Descriptive statistics of the FGD showed that among the seven experts from the TB center, two (28.6%) were in 31–40 years, three (42.8%) were in 41–50 years, and two (28.6%) were in 51–60 years old groups. Moreover, two (28.6%) had higher secondary and bachelor education each, and three (42.8%) had master level education. Only one female medical officer took part in FGD.

#### 4.2.1. General Conditions of the Health Facilities

The results of qualitative data (in-depth interviews, KIs and FGD) were summarized in the form of categories and sub-categories mentioned in Table 4.

General conditions of the health facilities were unsatisfactorily shown by the statements of patients during in-depth interviews, KIs and FGD.

Patients raised their concerns about DOTS facilities in different TB centers.

*“Safe drinking water is not available. We do not even have drinking water. I have been coming to this TB center since the last four months, but still no water is available at the center.”*

(Patient-A-11, age 34, female)

Safe drinking water was almost not available in all TB centers. Patients reported about the availability of water as follows:

*“I didn’t see any water coolers in the TB center. I brought water with me from home every time. However, I have seen people drink from the tap at the center.”*

(Patient-E-3, age 25, male)

This was one of the rural TB centers, where people were mostly using tap water to drink.

During a key-informant interview at the TB center:

*“We are in a temporary building, here, no water is available for drinking, and no wash rooms. Whenever there is need of water, we manage it from an outside building.”*

(KI-Medical Officer)

About 61% of the patients ranked availability of space as very low or low. Moreover, they shared their experiences and opinions with researchers and data collectors. All the patients, with their caretakers, were sitting together, which could also be a potential risk factor for tuberculosis.

*“There is no place for sitting at the center. Some space is available, but it is allocated to females. Usually, we stand for the whole day, waiting for doctor’s appointment. No chairs are there, at the center, too.”*

(Patient-A-44, age 45, male)

*“We have not enough space for patients in the TB center. Females are setting in front of the laboratory where everyone has to pass through them.”*

(KI-laboratory technician)

One major concern was the privacy of patients during treatment. About the privacy, the patients expressed their opinions as:

*“Whenever, I come to TB center, the doctors check many patients in the room. There is no privacy at all; even sometimes my own village people came to know that here I have TB.”*

(Patient-G-2, age 21, female)

*“Many problems I cannot discuss in front of the doctor, as many patients are always sitting around.”*

(Patient-H-1, age 28, female)

#### 4.2.2. DOTS Process

The quantitative data findings showed that 64% of the patients were satisfied from the process of DOTS. During an in-depth interview, a patient stated that:

*“We have no problem in the process of appointment and the health workers have very good behavior with patients.”*

(Patient A-34, age, 29, female)

#### 4.2.3. Outcome of DOTS

Most patients (>50%) were not satisfied with the effects of medication when they were asked about regaining energy after medication.

*“I have regained no energy, since I started these medicines, but of course it has reduced my cough.”*

(Patient-A-23, age 45, female)

*“It is very difficult to take these medicines. I feel very weak, have no energy and no mood to work and walk.”*

(Patient-C-139, age. 55, male)

#### 4.2.4. Other Issues

There were several issues that are linked with a low satisfaction level among the female patients in the study area. For example, females raised their concerns about the gender related issues as follows:

*“I cannot come alone to the TB center. If my husband is free, he will come with me or I will call my brother to take me to the health center.”*

(Patient-A-131, age 24, female)

*“Sometime, I feel pain, but have to wait for the male family member. When they are free, then we will go to see the doctor. I am not allowed to go out from home without being accompanied by someone from the family.”*

(Patient-F-3, age 27, female)

#### 4.2.5. Low Income and Poverty

Low income and poverty were also the reasons of patients’ low satisfaction level. A patient stated his opinion as follows:

*“I am a student, and my father is a wage laborer, who supports a family of ten. Every day, I get an amount from my father to go to college. Besides this, he has to pay me when I go to see the doctor, every month or two times in a month. The cost of the medicine and the traveling cost are very high. Usually, I don’t buy some of the medicine due lack of money.”*

(Patient-C-9, age 20, male)

Regarding the poverty of the patients, those patients who were poor having no homes stated that:

*“We are from the northern part, and every year we come here in winter and go back in the summer. We do not know someone to help us to approach a doctor. We are not treated the same way as the people from the district are.”*

(Patient-H-2, age 45, male)

During the FGD, the health care personnel agreed on the following issues that caused patients’ low level of satisfaction.

The main reason of the patients’ dissatisfaction is due to the unavailability of the basic facilities at the health care center. The main TB health center is situated in a temporary building. There is a facility for X-ray machines. The patients are asked to bring an X-ray from outside private laboratories which cost them more. Furthermore, the patients do not follow the instructions of doctors which resulted in less improvement in their health status (FGD).

## 5. Discussion

The World Health Organization, along with national governments, is committed to combatting tuberculosis, and Pakistan is no exemption. The National Tuberculosis Program (NTP) is trying to provide facilities for its patients. This study is very important because it evaluates patients’ perceptions and feelings towards the NTP steps and the facilities provided to them at TB centers in rural and urban areas. The study reveals different satisfaction levels in different aspects of the DOTS program. The majority of patients are satisfied with this program. However, within the satisfaction levels, the more highly satisfied category of patients were a very low percentage. Satisfaction is low in some items. For example, the availability of safe drinking water, space for patients, and privacy at TB centers. During the survey, most patients reported that safe drinking water was not available at their TB center. The clean drinking water can improve the health of the patients. For instance, Cardoso, et al. [49] reported from Brazil that drinking contaminated water by TB patients caused intestinal parasites.

The availability of space for patients is very crucial in the context of Pakistan, and other developing countries, for female patients particularly. Most patients were dissatisfied with availability of space. They shared their experiences and opinions with researchers and data collectors. All the patients, with their caretakers, were sitting together, which could also be a potential risk factor for tuberculosis. In addition, respondents were not satisfied with the privacy at the centers. In the doctor’s consultation room, 8–12 patients were sitting together, being checked by the doctor. Participants raised this issue in IDI and focus group discussions. The main district TB center was located in a temporary building which caused the patients dissatisfaction. The findings of this study are consistent with Onyeonoro, et al. [28]. They revealed that in Southern Nigeria, patients’ low satisfaction with facilities at TB centers is because of the public DOTS facilities were neglected and lacked basic services, needed for the comfort of patients. Therefore, patients’ comfort is not only needed in Pakistan, but also in other countries. Contrary to our findings, Panezai, et al. [50] reported that patients were satisfied with the privacy at health care facilities in Balochistan, Pakistan.

In the process of medication, patients were also found to be not satisfied with the waiting time item, convenient service hours, and the process for appointments. Patients were spending the whole day in a TB center for a single appointment. In most TB centers, there was only one doctor, who was checking the number of patients with various diseases, particularly at RHCs. At the main center, there was also only one doctor checking the patients. Although TB services were readily accessible, items such as satisfaction on access to collect medicine, availability of medicine, and laboratory services were high, yet the patient satisfaction score for waiting time was low. A study conducted by Nwabueze, et al. [51] in Nigeria on HIV/AIDS patients revealed that a low satisfaction with the waiting time is because of the multiple visits (at least three visits) required before commencing treatment. Another reason for low satisfaction with the waiting time is the fact that most patients were young and were at an economically productive age. Being engaged in economic activities, they were the breadwinners of the family. Therefore, spending such a long time in TB centers results in high opportunity costs to them, which leads to dissatisfaction. Onyeonoro, et al. [28] also revealed that patients were not satisfied with the waiting time at TB centers, and opportunity costs in this context were the earnings lost, consequently upon the time spent while accessing TB care. Similarly, our findings are consistent with the findings of [52], who reported that a long waiting time causes delays in getting care.

In many scoring items, patients were more highly satisfied with the DOTS strategy in the district. The findings of this study confirm earlier studies in that most patients were found to be satisfied with the treatment strategies. For example, Nezenega and Tafere [41] and Getahun and Nkosi [40] revealed that in Ethiopia, 90% and 67% of patients were satisfied, respectively. Likewise, according to Gupta [53] in India and Chimbindi, et al. [30] in KwaZulu-Natal, most patients were satisfied with TB health services.

Limited studies have discussed the effect of patients’ socio-economic characteristics on their satisfaction level in Pakistan. The findings of this study show that except in the age category, all variables have significant association with satisfaction levels. Our results are also inconsistent with some findings of previous studies. For instance, Onyeonoro, et al. [28] revealed that young patients were comparatively more satisfied than older patients in their study. However, Schoenfelder, et al. [54] reported that older patients were more satisfied than younger patients. Gender was found to be significant with the satisfaction level of patients from TB facilities. Male patients were more satisfied than females in our study. Males have comparatively less problems and were more educated and employed. They could talk and knew how to get appointments at the TB centers. In addition, men could come to urban TB centers more easily than women due to socio-cultural constraints on the latter, which was the reason that men were more satisfied than women. There were also comparatively more facilities in urban centers than rural centers. Similar findings were reported by Onyeonoro, et al. [28]. They revealed that men scored higher in satisfaction than women.

Marital status was also one of the important characteristics that influenced patients’ satisfaction. The findings show that married patients were more satisfied than their unmarried counterparts. Married people receive more support from their families and in the case of females from husbands, and could come to the TB centers more easily. Moreover, in Pakistan’s context, most women patients cannot go to health centers alone. They must be accompanied by another person as a caretaker. One reason is that the women were mostly living in homes as a housewife; they were not exposed to the external environment. Therefore, they need someone who knows the hospital and location of the health center. Another reason is that there are cultural constraints on women; they are not allowed to go out alone from the home. Hence, married women have the support of the husbands, they were older age. Consequently, married patients were more satisfied than unmarried. The finding of the study are in agreement with Onyeonoro, et al. [28], who reported that in Southern Nigeria, married patients had higher scores for satisfaction than unmarried. However, our findings are in contrast with the findings of Quintana, et al. [29] and Chimbindi, et al. [30], which revealed that unmarried satisfaction was higher than married.

Patients from urban areas were more satisfied with their TB centers than from rural. Patients from rural areas had several hurdles and problems in coming to urban TB centers. Hence, the patients living in urban areas were more satisfied with TB health centers compared to rural patients. As nearly 50% of the world’s population lives in rural areas, but less than 38% of nurses and less than a quarter of physicians work in these areas [55]. Similarly, in Pakistan, only 33% of the population lives in the range of 5 km from a health facility [56]. There is a strong urban bias in Pakistan in terms of establishment of health facilities and practice of doctors [57,58]. Most of the higher level health facilities, about 78%, had female doctors, as compared to only 28% primary level facilities in Punjab and Khyber Pakhtunkhwa [59]. Therefore, this has resulted into urban biasness in the health care system. Similar findings were reported by Mohamed, et al. [24], who reported that in Sudan, 66% from urban patients and 48.9% from rural patients were satisfied with their TB treatment facilities.

Literate patients had more knowledge and understood the signage and directions to TB centers. They knew how to talk and interact in a better way with health professionals. Moreover, they were less dependent on health facilities. For example, literate patients can read posters and pamphlets. Therefore, they were more satisfied than the illiterate patients. Our results confirm the findings of Onyeonoro, et al. [28] and Mohamed, et al. [24], who revealed that higher educational status leads to higher levels of satisfaction, more likely to appreciate information and services provided by DOTS staff, as well as making better informed decisions to improve their health. However, these findings of the study are in contrast with Rahmqvist and Bara [60] and Getahun and Nkosi [40], who revealed that in Sweden and Ethiopia, highly educated patients are less likely to be satisfied due to their high expectations, and tend to be less satisfied with health care services.

In our analysis, patients who were employed were more satisfied than the unemployed. A significant number of patients were less than twenty years of age or housewives; they were dependent on their parents or husbands. It was difficult for them to finance their health expenditures, which leads to their low level of satisfaction with the DOTS strategy in the district.

The patients who had good economic background were more satisfied than poor. For example, those living in their own houses had a higher satisfaction than the other patients. House owners were mostly district residents, whereas the non-owners were from the northern areas. They were seasonal migrants, living in rented houses and slums. They were living in poor economic conditions, which led to their dissatisfaction with TB services.

The patients who were comparatively rich and could finance such expenditures, have a higher level of satisfaction. Hence, in the main household occupation, servants were higher in satisfaction than households’ dependent on agriculture. Likewise, income level plays a significant role in patients’ satisfaction with DOTS [40]. Our findings show that patients in the lowest category and the highest category of income were not satisfied with TB services; however, patients from middle class families were more satisfied. The patients belonging to the very low-income group had several socio-economic constraints that led to their lower satisfaction. For example, they could not finance their medical expenditures and transportation cost. Our findings are consistent with Charokar and Jain [61], who reported that in India, higher income patients were more satisfied than lower income patients.

This study has incorporated different data collection techniques. The data have been collected at all TB centers in the district. Samples from every TB center have been incorporated in the data analysis. Satisfaction on different aspects was included in the study, and the data were integrated from different sources. The outcome of the study can be generalized to other parts of the province and country.

### Limitations of the Study

This study is cross-sectional and not longitudinal, where a patient’s satisfaction may change over time. There could be a possibility of potential selection bias, particularly in the in-depth interviews. The study could not incorporate the confirmatory factor analysis due to its small sample size and having only validated the questionnaire on a single parameter. The study will be enhanced if confirmatory factor analysis is performed and tested on a large sample size.

## 6. Conclusions

The majority of patients were satisfied with the DOTS delivery process. The study has very interesting findings regarding the satisfaction of patients. Patients from rural areas, particularly females, are confronted with many socio-cultural problems that reduced their satisfaction level. Moreover, the poor and lower economic status patients were not satisfied. The study has several implications for policymakers and individual households. At health centers, doctor availability and reduced waiting time should be addressed, which would enhance DOTS facilities. Moreover, female health workers should be involved in the DOTS program, so that female respondents can receive their medication at their doorstep. Improving the infrastructure facilities at TB centers, and allocation of doctors and nurses at the rural centers, would have a positive impact on the DOTS strategy. Consequently, it would also improve patients’ adherence. Researchers in this area are encouraged to incorporate patients’ satisfaction with their adherence to DOTS in the study area, which we could not incorporate in our study.

## Figures and Tables

**Table 2:** Patient comparison by group.

Variables	Satisfaction			Significance	
	Dissatisfied	Satisfied		Chi-Square Test Value	*p*-Value
Gender (X1)					
Female	61 (43.88)	78 (56.12)	7.639		0.010 **
Male	36 (27.69)	94 (72.31)			
Marital status (X2)					
Unmarried	48 (52.75)	43 (47.25)	16.716		0.000 **
Married	44 (27.16)	118 (72.84)			
Deceased wife	5 (31.25)	11 (68.75)			
Location (X3)					
Rural	26 (25.74)	75 (74.26)	7.466		0.010 **
Urban	71 (42.26)	97 (57.74)			
Employment status (X4)					
No	82 (48.81)	86 (51.19)	31.548		0.000 **
Yes	15 (14.85)	86 (85.15)			
House ownership (X5)					
No	72 (47.68)	79 (52.32)	20.168		0.000 **
Yes	25 (21.19)	93 (78.81)			
Literacy (X6)					
Illiterate	62(42.47)	84(57.53)	5.683		0.020 *
Literate	35 (28.46)	88 (71.54)			
Main households’ occupation (X7)					
Agriculture	35 (45.45)	42 (54.55)	14.30 †		0.000 **
Trade	19 (46.34)	22 (53.66)			
Labor	31(33.70)	61 (66.30)			
Servant	4 (12.12)	29 (87.88)			
Others	8 (30.77)	18 (69.23)			
Households’ level of Income (X8)					
<250 USD	68 (46.26)	79 (53.74)	14.44 †		0.000 **
250–500	20 (24.10)	63 (75.90)			
501–750	5 (21.74)	18 (78.26)			
>750	4 (25.00)	12 (75.00)			
Age categories (X9)					
15–20 Years	29 (46.03)	34 (53.97)	7.65 †		0.1
21–30	29 (32.95)	59 (67.05)			
31–40	17 (31.48)	37 (68.52)			
41–50	19 (42.22)	26 (57.78)			
51–60	3 (15.79)	16 (84.21)			

Source: Field Survey, 2016–17; ** Significance levels: *p* ≤ 0.01; * Significance level: *p* ≤ 0.05, † shows Fisher-exact test.

**Table 3:** Results of the binary logistic regression.

	COR (95% CI)	*p*-Value	AOR (95% CI)	*p*-Value
Gender (X1)				
Female	Ref.		Ref.	
Male	2.04 (1.23–3.40)	0.006 **	2.21 (1.07–4.58)	0.033 *
Marital status (X2)				
Unmarried	Ref.		Ref.	
Married	2.57 (1.51–4.36)	0.000 **	3.126 (1.45–6.73)	0.004 **
Deceased wife	2.20 (0.71–6.81)	0.172	2.524 (0.47–13.68)	0.283
Location (X3)				
Rural	Ref.		Ref.	
Urban	2.11 (1.23–3.63)	0.007 **	1.89 (0.93–3.69)	0.078 *
Employment status (X4)				
No	Ref.		Ref.	
Yes	5.45 (2.92–10.23)	0.000 **	5.22 (2.44–1.21)	0.000 **
House ownership (X5)				
No	Ref.		Ref.	
Yes	3.39 (1.97–5.85)	0.000 **	3.82 (1.94–7.54)	0.000 **
Literacy (X6)				
Illiterate	Ref.		Ref.	
Literate	1.86 (1.11–3.09)	0.018 *	2.17 (1.11–4.25)	0.023 *
Main households’ occupation (X7)				
Agriculture	Ref.		Ref.	
Trade	0.97 (0.45–2.06)	0.927	0.59 (0.22–1.59)	0.300
Labor	1.64 (0.88–3.06)	0.120	1.22 (0.56–2.65)	0.618
Servant	6.04 (1.94–18.85)	0.002 **	4.42 (1.12–17.38)	0.033 *
Others	1.86 (0.73–4.83)	0.193	0.63 (0.18–2.16)	0.459
Households’ level of Income (X8)				
<250 USD	Ref.		Ref.	
250–500	2.71(1.49–4.93)	0.001 **	2.39 (1.13–5.04)	0.023 *
501–750	3.10 (1.09–8.79)	0.033 *	1.67 (0.42–6.63)	0.463
>750	2.58 (0.79–8.38)	0.114	1.33 (0.33–5.35)	0.693
Age categories (X9)				
15–20 Years	Ref.		Ref.	
21–30	1.79 (0.93–3.48)	0.083	0.96 (0.41–2.24)	0.924
31–40	1.81 (0.859–3.83)	0.118	0.79 (0.27–2.35)	0.678
41–50	1.12 (0.51–2.42)	0.784	0.66 (0.22–1.92)	0.445
51–60	15.88 (1.99–126.20)	0.009 **	7.39 (0.73–74.88)	0.090
Hosmer and Lemeshow Test	Chi-Square Value = 11.017, df = 8 and *p* = 0.201			

Source: Field Survey, 2016–17; ** Significance levels: *p* ≤ 0.01; * Significance level: *p* ≤ 0.05.

**Table 4:** Categories and sub-categories identified during qualitative data collection.

Categories	Sub-Categories
General conditions of health facilities	Infrastructure facilities
	Availability of health services and facilities
DOTS process	Convenience
	Process of appointment
	Attitude of the health personnel
DOTS outcome	Improvement in health status
	Psychological effects
Other issues	Gender related
	Rural and urban
	Low income and poverty

## Data Availability

Data will be shared on email upon suitable request.

## Appendix A

**Table A1:** Measuring Items.

Overall Satisfaction 1 Item
Satisfaction in Structure of DOTS
Space for Patients
Availability of Safe Drinking Water
Availability of Toilets
Treatment Process
Access to Medicine
Privacy at TB Centre
Laboratory Services
Availability of Medicines
Availability of Doctors
Availability of Equipment
Availability of Signage / Directions
DOTS Processes
Convenience of Service Hours
Waiting Time
Appointment Process
Attitude of Health Personnel
Care of Health Personnel
Guidance on Sickness
Consultation
Skill of Health Professionals
Cost of Medicine
Cost of Transportation
Equality between Rich and Poor
Outcome of DOTS
Symptoms Reduction
Overall effect of Medicine
Psychological Effect
Feeling Energetic
